# Supplementary material for: Transient marine euxinia at the end of the terminal Cryogenian glaciation
Source: Nat Commun. 2018 Aug 1;9:3019. doi: 10.1038/s41467-018-05423-x (PMC6070556; doi:10.1038/s41467-018-05423-x)
Supplement: Supplementary file 1 — Supplementary Information [file 41467_2018_5423_MOESM1_ESM.pdf]

Supplementary Information

Transient marine euxinia at the end of the terminal Cryogenian glaciation

Lang et al.

Supplementary Note 1

Geological back ground

The terminal Cryogenian (Marinoan) glacial deposit in the Yangtze Block is represented by the Nantuo Formation, which is dated between 654 and 635 Ma[1-4]. The Nantuo Formation is widely distributed throughout the Yangtze Block, and conformably underlies the cap carbonate of the basal Doushantuo Formation, but variably overlies different strata depending on the depositional environments (Supplementary Fig.1). In the inner shelf depositional environment, the Nantuo Formation unconformably overlies the terrestrial/shallow marine sandstone of the Liantuo/Chengjiang formations, whereas it conformably overlies the Datangpo and Fulu formations in the outer shelf, slope, and basinal settings. The thickness of the Nantuo Formation increases from 0-100 m in the inner shelf to >1500 m in the basinal successions[3,5]. The Nantuo Formation is composed of one or multiple diamictite-siltstone/sandstone cycles[5]. In general, there is a decreasing trend of sandstone/siltstone contents from deep to shallow water settings. In the inner shelf facies, the Nantuo Formation is mainly composed of massive diamictite with sporadic occurrences of thin siltstone/mudstone layers, whereas in the deeper slope and basinal environment, it is composed of alternating depositions of diamictite and siltstone units.

Abundant pyrite concretions are discovered near the top of Nantuo Formation (Supplementary Fig. 2), particularly in the upper most 0.5 to 10 m of the top diamictite unit that underlies the siltstone/mudstone layer (Supplementary Fig.2).

Supplementary Note 2

1D-DAR model

Model parameters and results

In order to quantify $\delta^{34}S_{py}$ and pyrite content, the following parameters: D_s, s, R, $[SO4]_0$, $\delta^{34}S_{sw}$ and α need to be determined. The default parameters are listed in Supplementary Table 4.

D_s is the diffusion coefficient in sediments, and can be calculated by the following equation:

$$D_s = \frac{D_{sw}}{1-\ln(\emptyset^2)} \quad (1)$$

where D_{sw} is the diffusion coefficient in seawater and is temperature dependent, varying between 4.64×10^{-6} cm^2 s^{-1} and 9.95×10^{-6} cm^2 s^{-1} from 0 °C to 25 °C[8]. At a porosity of 60%, D_s varies from 2.29×10^{-6} cm^2 s^{-1} to 4.92×10^{-6} cm^2 s^{-1} from 0 °C to 25 °C. Here we choose the intermediate value of 3.61×10^{-6} cm^2 s^{-1} for D_s.

The sedimentation rate (s) varies between ~0.005 cm yr^{-1} and ~0.2 cm yr^{-1} in modern siliciclastic continental margin area[8]. Here we choose 0.01 cm yr^{-1} as the value of s in this model.

R is the first-order rate constant of sulfate reduction within porewater. Both temperature and organic substrate affect R[10]. High organic substrate availability and moderate temperature (~25 °C) could produce a high value of R that can reach as high as ~20 yr^{-1} [10]. Here we choose 1 yr^{-1} as the default value.

In addition to above parameters, pyrite sulfur isotope and content also relate to $[SO4]_0$, $\delta^{34}S_{sw}$ and α. $[SO4]_0$ is the seawater sulfate concentration during the meltdown of Marinoan Snowball Earth. $\delta^{34}S_{sw}$ represents the sulfur isotope of sulfate at the seawater-sediments interface. Here, according to previous estimation, we set $[SO4]_0$ and $\delta^{34}S_{sw}$ at values of 3 mM L^{-1} and +30‰, respectively[7,9]. The fractionation factor (α) is set at 0.96[10].

Assuming DSR initiated at the water-sediment interface (WSI), pyrite sulfur isotope and content cross plot can be modified by using the aforementioned parameters as listed in Supplementary Table 4. The modeling results show that pore water sulfate could be consumed completely at the depth ~0.8 m below WSI and pyrite content reaches the maximum value of 6.99 vol.% (Fig. 3c). Although the instantaneous value of $\delta^{34}S_{py}$ could up to > +100‰, the

maximum value of cumulative $\delta^{34}S_{py}$ is ~ +8‰.

Sensitivity test

In order to test the sensitivity of D_s, s, R and $[SO4]_0$, we initiate the default values and modify each single parameter. Both the cumulative $\delta^{34}S_{py}$ and pyrite content are quantified in each run. We varied the D_s between 2.29×10^{-6} cm^2 s^{-1} and 4.92×10^{-6} cm^2 s^{-1}. Sedimentation rate is allowed to vary between 0.005 cm yr^{-1} and 0.2 cm yr^{-1} and R is allowed to change from 0.1 yr^{-1} to 10 yr^{-1}. $[SO4]_0$ is set varying from 0.5 mM L^{-1} to 5 mM L^{-1}.

The modeling results indicate that pyrite content is controlled by D_s, s, R and $[SO4]_0$, whereas the cumulative $\delta^{34}S_{py}$ is not sensitivity to these parameters (Supplementary Figs. 5-8).

1D-DAR model with limited organic matter

Model description

Equation 4 in the Method part describes DSR with unlimited supply of organic matter, i.e. pyrite formation is controlled by seawater sulfate alone. This is an idealized situation. In fact, availability of organic matter also controls pyrite formation in sediment. The modified 1D-DAR model can be expressed by the following equation:

$$\frac{\partial[^{i}SO4]}{\partial t} = D_s\left(\frac{\partial^2[^{i}SO4]}{\partial z^2}\right) - s\left(\frac{\partial[^{i}SO4]}{\partial z}\right) - R_{3i}[^{i}SO4][CH2O] \quad (2)$$

Where [CH2O] represent organic matter content in sediments. Because organic matter is continuously consumed by SRM in sediment before it is completely consumed or porewater sulfate concentration decreases to zero, [CH2O] decreases through depth. The equation for [CH2O] profile can be expressed by:

$$\frac{\partial[CH2O]}{\partial t} = -R_{32}[^{32}SO4][CH2O] - R_{34}[^{34}SO4][CH2O] \quad (3)$$

Because equation 2 is a non-linear equation, it does not have an analytical solution. We use Mat-Lab software to simulate the pyrite formation processes.

Parameter setting and results

By using the default parameters used in the 1D-DAR model (D_s, s, R and $[SO4]_0$ are 3.61×10^{-6} cm^2 s^{-1}, 0.01 cm yr^{-1}, 1 yr^{-1} and 3 mM L^{-1}), [CH2O] is assigned a series of number,

ranging from 0 wt.% to 10 wt.%. The modeling results indicates that pyrite content increase with the increasing of organic matter content, whereas pyrite sulfur isotopes decrease with the decreasing of organic matter (Supplementary Fig. 9).

Rayleigh distillation model

Model parameters and results

In the Rayleigh distillation model, pyrite content and the cumulative $\delta^{34}S_{py}$ are related to the following parameters: $\delta^{34}S_{sw}$, α, Φ, f, and [SO4]. Supplementary Table 5 summarizes the used parameters in the Rayleigh distillation model. The value of $\delta^{34}S_{sw}$ in the aftermath of Marinoan snowball Earth meltdown is estimated at +30‰[6,7]. The fraction factor (α) varies from 0.96 to 0.98. The porosity (Φ) of coarse-grained sediments varies between 45% and 60%[8]. Here, we choose 60% as the value of Φ for making the maximum estimation. f varies from 0 to 1. Seawater sulfate concentration ([SO4]) is set at 3 mM L^{-1} [9].

By assigning the aforementioned parameters, both pyrite content and the cumulative $\delta^{34}S_{py}$ can be calculated. The Rayleigh distillation modeling results indicate that a closed porewater system can only account for < 0.1 vol.% of pyrite content (Fig. 3a). The $\delta^{34}S_{py}$ values are sensitivity to α and f (Fig. 3a and d).

1D-DR model

Model parameters and results

In the 1D-DR model, the cumulative $\delta^{34}S_{py}$ and pyrite content are related to the follow parameters: D_s, R, $\delta^{34}S_{H2S}$, Δ_{py} and $[SO4]_0$. Supplementary Table 6 summarizes the used parameters in this simulation. The modeling results indicate that the $\delta^{34}S_{py}$ is controlled by the $\delta^{34}S_{H2S}$ in sulfidic seawater, while pyrite content is sensitive to other factors except for $\delta^{34}S_{H2S}$.

Sensitivity test

To test the sensitivity of the parameters, D_s is allowed to vary between 4.89×10^{-6} cm^2 s^{-1} and 7.96×10^{-6} cm^2 s^{-1}, corresponding to the temperature range from 0 °C to 25 °C, R is allowed to range from 0 to 1000, and $\delta^{34}S_{H2S}$ is allowed to vary between -10‰ and +30‰. The sensitivity test suggest that the $\delta^{34}S_{py}$ is not affected by these parameters (Supplementary Fig. 10).

Supplementary figures and tables

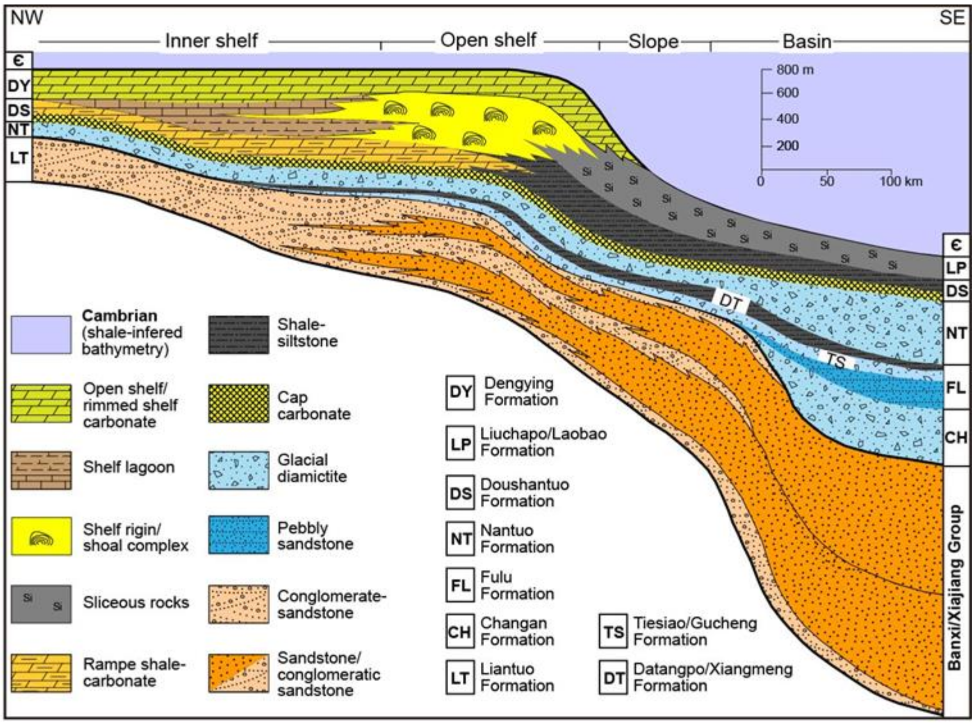

South China		Shallow water	Slope		Basin	
€	542Ma	Dengying Formation	Dengying Formation		Laobao/Liuchapo Formation	
Ediacaran	551Ma	Doushantuo Formation	Doushantuo Formation		Doushantuo Formation	
Cryogenian	635Ma	Nantuo Formation	Nantuo Formation		Nantuo Formation	
	654Ma		Datangpo Formation		Fulu Formation	
	663Ma		Gucheng/Tiesiao Formation		Changan Formation	
Tonian	715Ma	Liantuo Formation	Banxi Group	Xiajiang Group	Banxi Group	Xiajiang Group

Supplementary Figure 1: The Neoproterozoic stratigraphic framework and depositional model of the Yangtze Block[4].

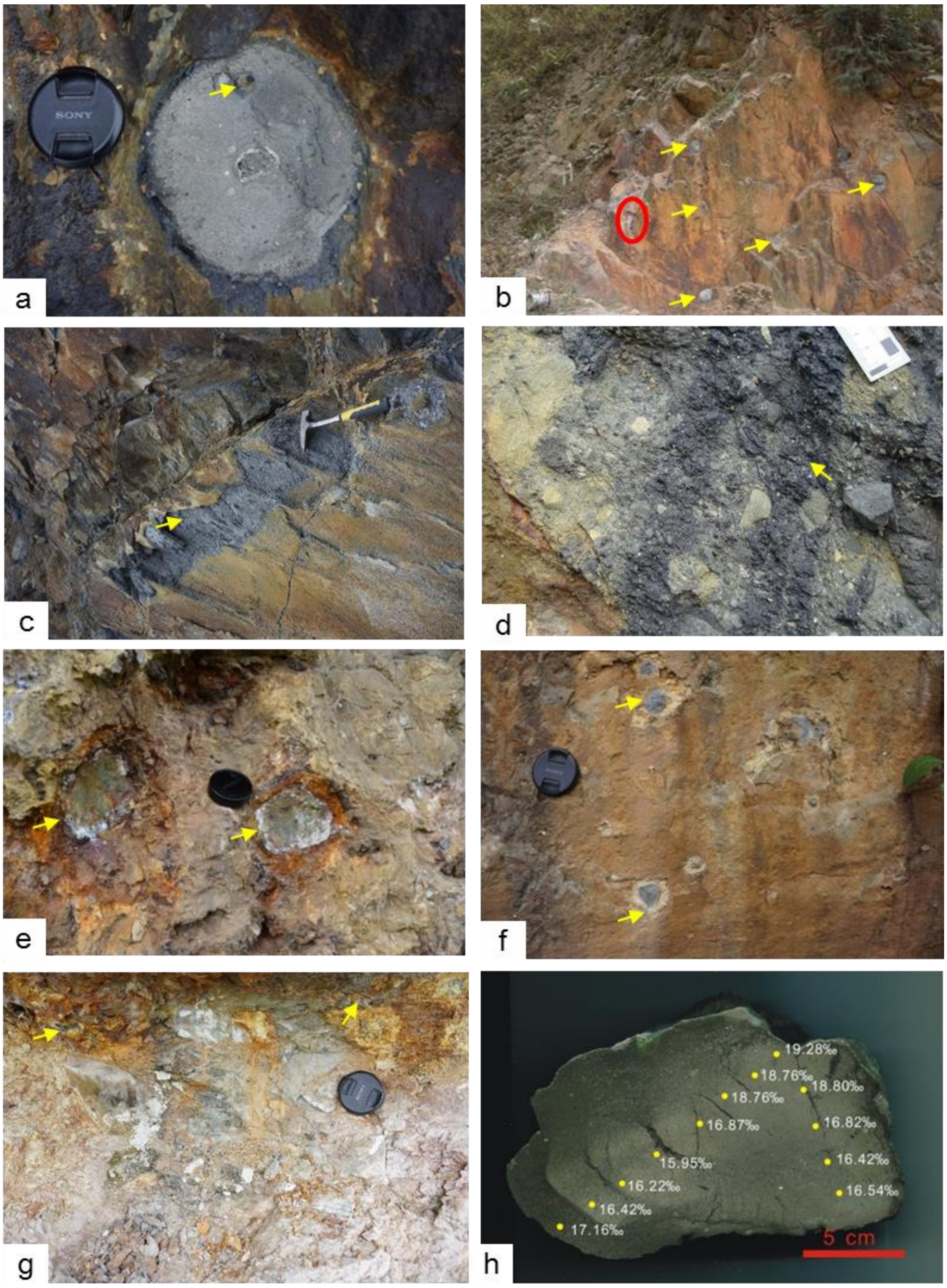

Supplementary Figure 2: Photographs of pyrite concretions in the field and in hand specimens. (a): Pyrite concretion in the Yangxi section, Sanjiang County, Guangxi Province, yellow arrow indicates siliciclastic clasts within the nodule. (b): Pyrite concretions (yellow arrows) in the Tongle section, Sanjiang County, Guangxi Province. (c): A massive pyrite aggregate (yellow arrow) in the Yangxi section, Sanjiang County, Guangxi province. (d): Black stratified massive diamictite in the Silikou section, Sanjiang County, Guangxi Province. (e): Pyrite concretions (yellow arrow) recorded in the cap carbonate in the Taoying section, Guizhou province. (f): Pyrite concretions (yellow arrow) in the Huakoushan section, Jiangkou County, Guizhou Province. (g): Small pyrite concretion (yellow arrows) in the Youxi section, Changyang County, Hubei Province. (h): Pyrite concretion from the Tongle section showing limited variations in $\delta^{34}S_{py}$.

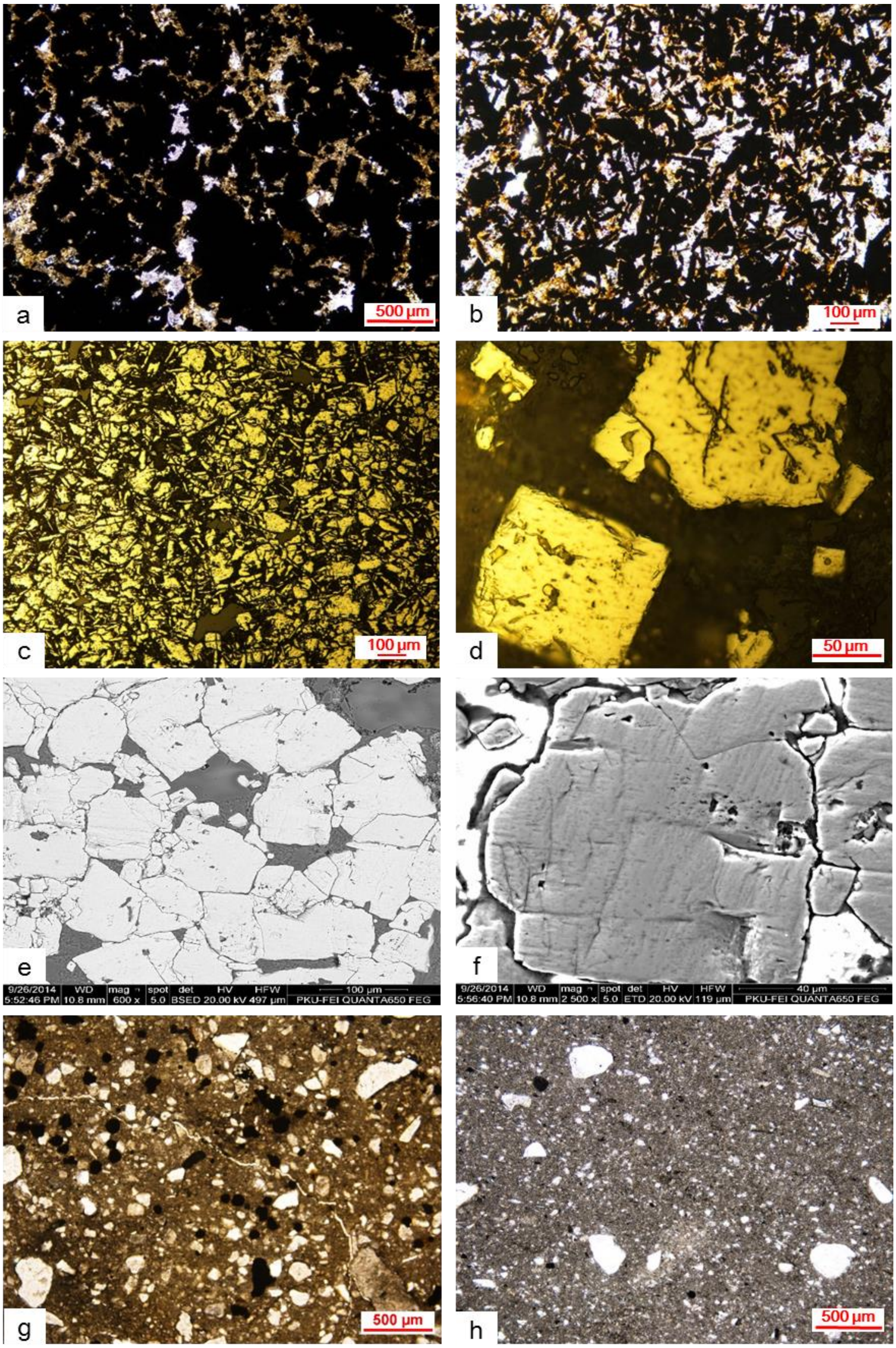

Supplementary Figure 3: Photomicrographs of pyrite concretions. (a) and (b): Under plain light, pyrites are opaque minerals, the transparent minerals are silica cements; dark brown minerals are fine sands/silts/clays. (c) and (d): Under reflected light, pyrite crystals show golden color. (e) and (f): BSE photomicrographs of pyrite, indicating the absence of framboidal cores. (g) and (h) Disseminated pyrites in the uppermost of Nantuo Formation in the Bahuang section and a drill core in Jiangkou County, respectively.

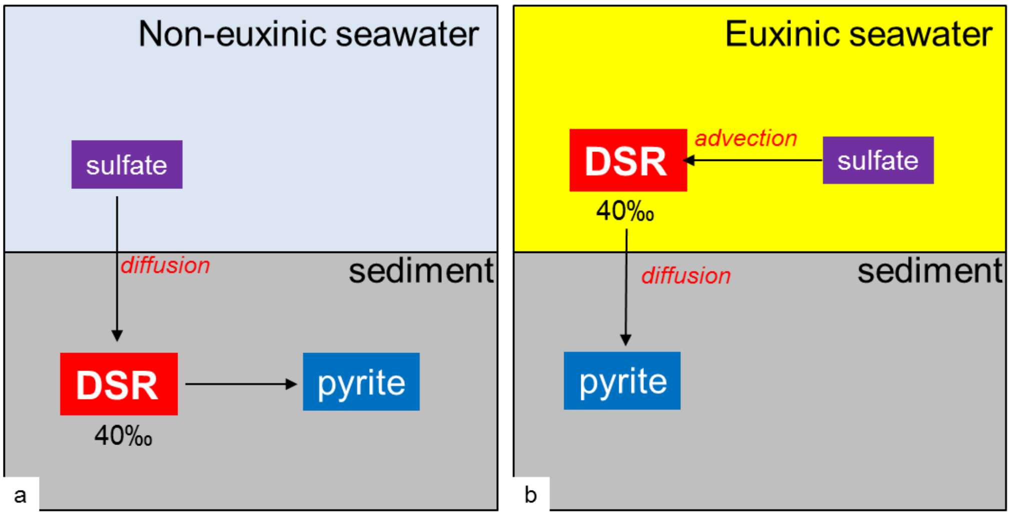

Supplementary Figure 4: Model structures of syndepositional pyrite formation in the open system. (a) DSR occurring within sediment with sulfate diffusion from seawater; (b) DSR occurring within seawater with H_2S diffusion into sediments.

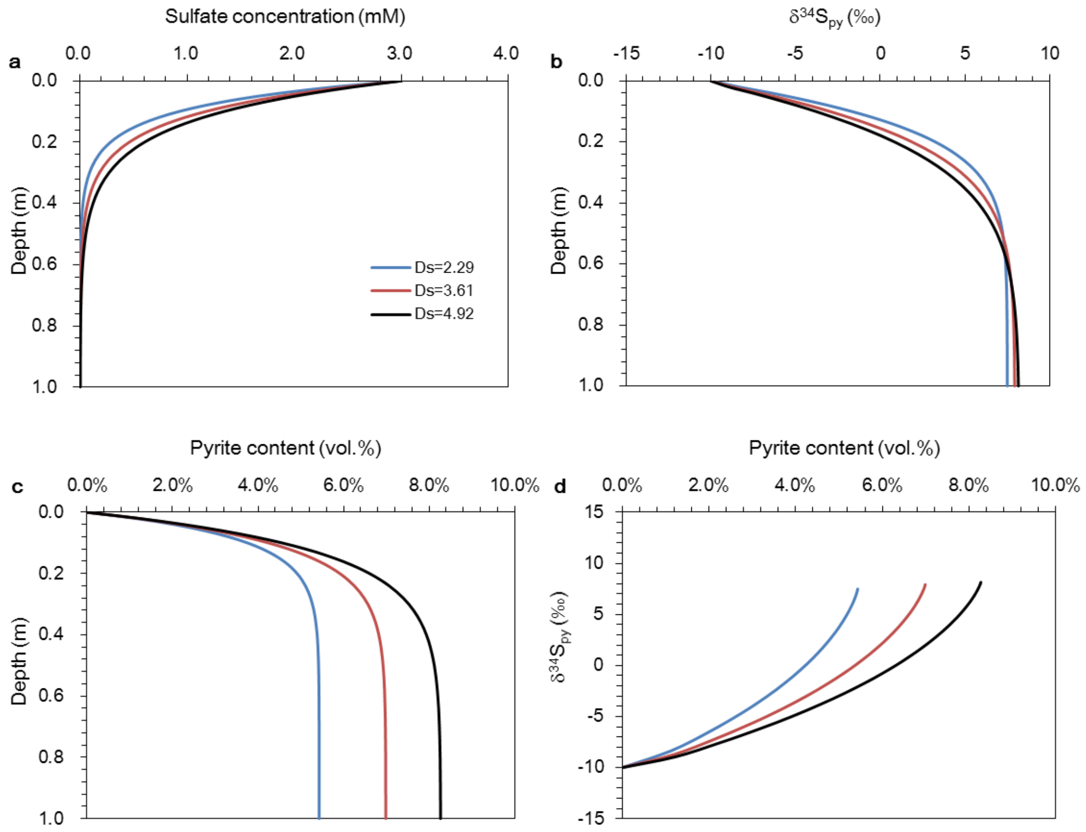

Supplementary Figure 5: Sensitivity test of D_s for the 1D-DAR model. D_s varies between 2.29×10^{-6} cm^2 s^{-1} and 4.92×10^{-6} cm^2 s^{-1}. (a) and (b): Variation of D_s only leads to a slightly change in pore water sulfate concentration and $\delta^{34}S_{py}$. (c): Pyrite content increase from 5.43% to 8.26% when D_s varies between 2.29×10^{-6} cm^2 s^{-1} and 4.92×10^{-6} cm^2 s^{-1}. (d): Cross plot showing the relationship between $\delta^{34}S_{py}$ and pyrite content by varying D_s. The default parameters for s, R and $[SO4]_0$ are 0.01 cm/yr, 1 yr^{-1} and 3 mM L^{-1}, respectively.

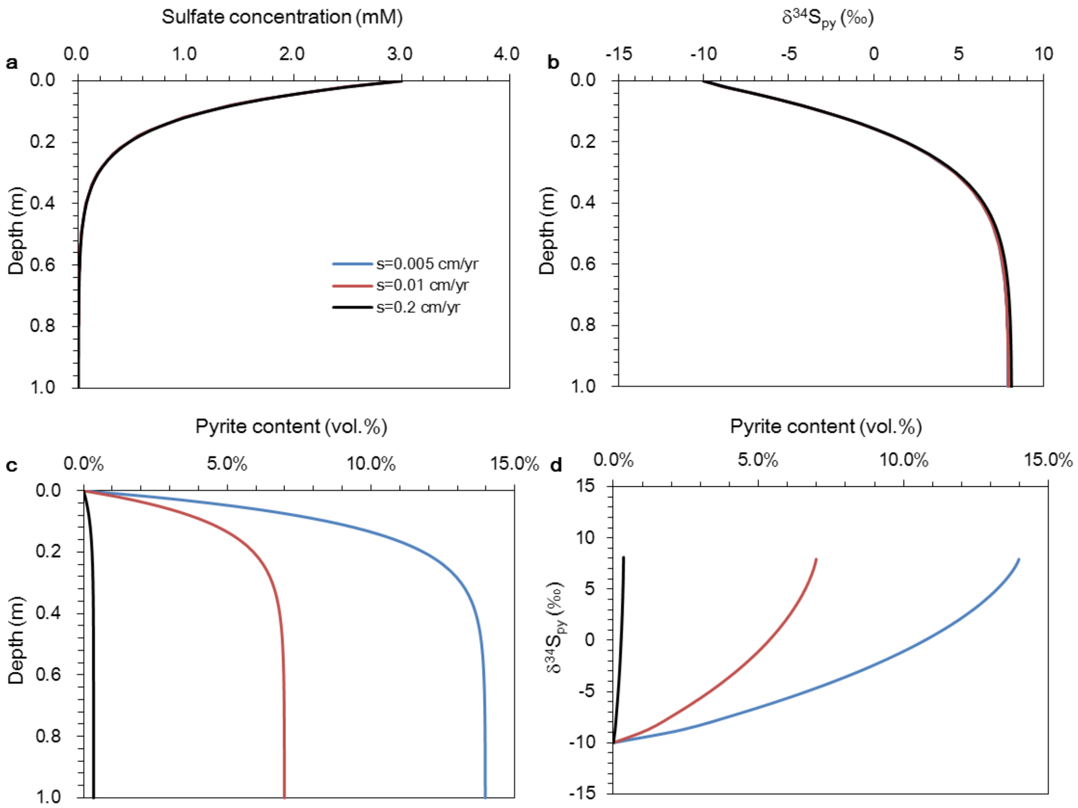

Supplementary Figure 6: Sensitivity test of the sedimentation rate for the 1D-DAR model. s varies between 0.005 cm yr^{-1} and 0.2 cm yr^{-1}. (a) and (b): Variation of s leads to negligible changes in both pore water sulfate concentration and $\delta^{34}S_{py}$. (c): Pyrite content is significantly affected by s. (d): Cross plot showing the relationship between $\delta^{34}S_{py}$ and pyrite content by varying s. The default parameters for D_s, R and $[SO4]_0$ are 3.61×10^{-6} cm^2 s^{-1}, 1 yr^{-1} and 3 mM L^{-1}, respectively.

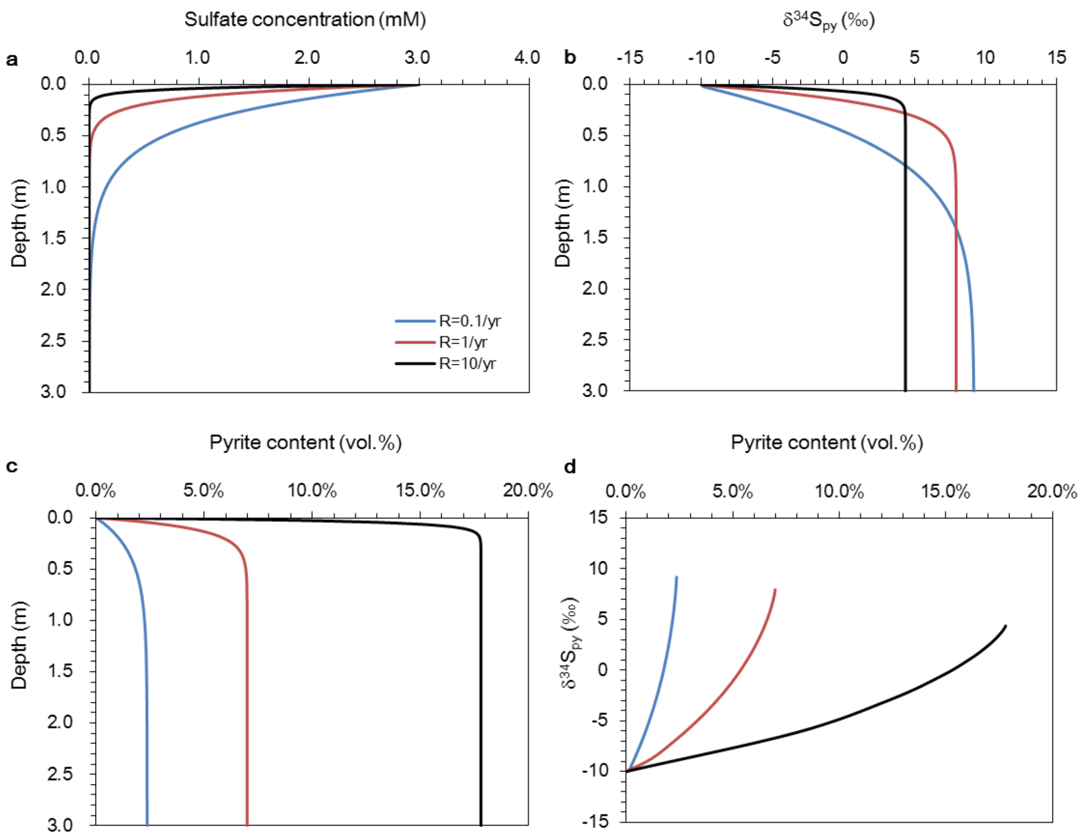

Supplementary Figure 7: Sensitivity test of the R for the 1D-DAR model. R varies from 0.1 yr^{-1} to 10 yr^{-1}. (a): Variation of R results in moderate changes in pore water sulfate concentration profiles. (b): Varying R from 0.1 yr^{-1} to 10 yr^{-1} leads to ~ 5‰ changes in the $\delta^{34}S_{py}$. (c) Pyrite content is significantly affected by R. (d): Cross plot showing the relationship between $\delta^{34}S_{py}$ and pyrite content by varying R. The default parameters for D_s, s and $[SO4]_0$ are 3.61×10^{-6} cm^2 s^{-1}, 0.01 cm yr^{-1} and 3 mM L^{-1}, respectively.

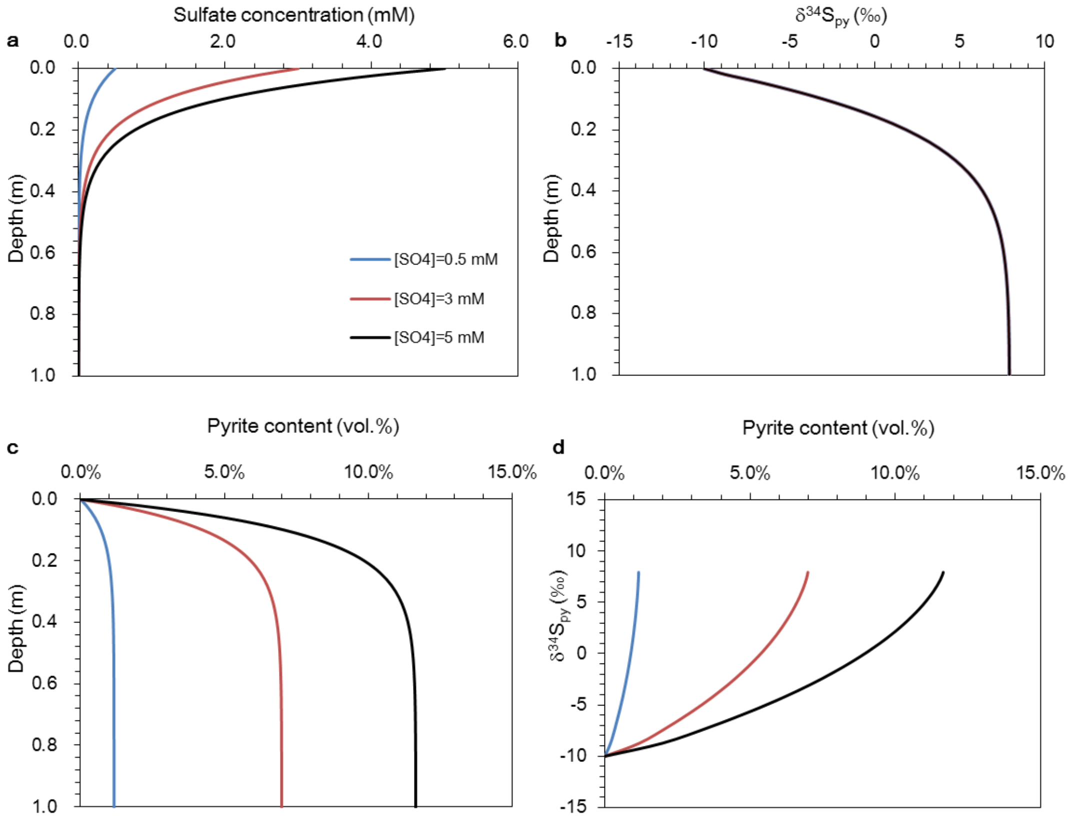

Supplementary Figure 8: Sensitivity test of the $[SO4]_0$ for the 1D-DAR model. $[SO4]_0$ varies between 0.5 mM L^{-1} and 5 mM L^{-1}. (a): Pore water sulfate concentration profile is significantly affected by $[SO4]_0$. (b): $\delta^{34}S_{py}$ is not affected by $[SO4]_0$. (c) Varying $[SO4]_0$ from 0.5 mM L^{-1} to 5 mM L^{-1} leads to pyrite content increasing from 1.16% to11.64%. (d): Cross plot showing the relationship between $\delta^{34}S_{py}$ and pyrite content. The default parameters for D_s, s and R are 3.61×10^{-6} cm^2 s^{-1}, 0.01 cm yr^{-1} and 1 yr^{-1}, respectively.

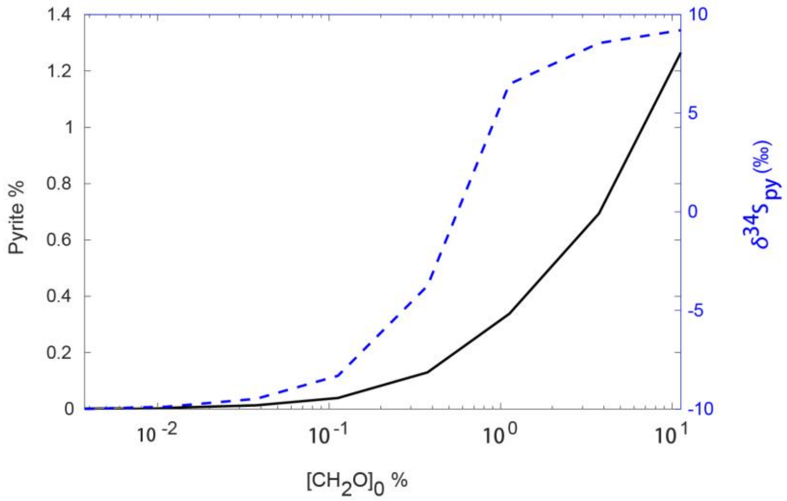

Supplementary Figure 9: The modeling results of 1D-DAR model with limited organic matter supply. The default parameters for D_s, s, R and $[SO4]_0$ are 3.61×10^{-6} cm^2 s^{-1}, 0.01 cm yr^{-1}, 1 yr^{-1} and 3 mM L^{-1} respectively. The plot showing a decrease in pyrite $\delta^{34}S$ with decreasing of available organic matter content.

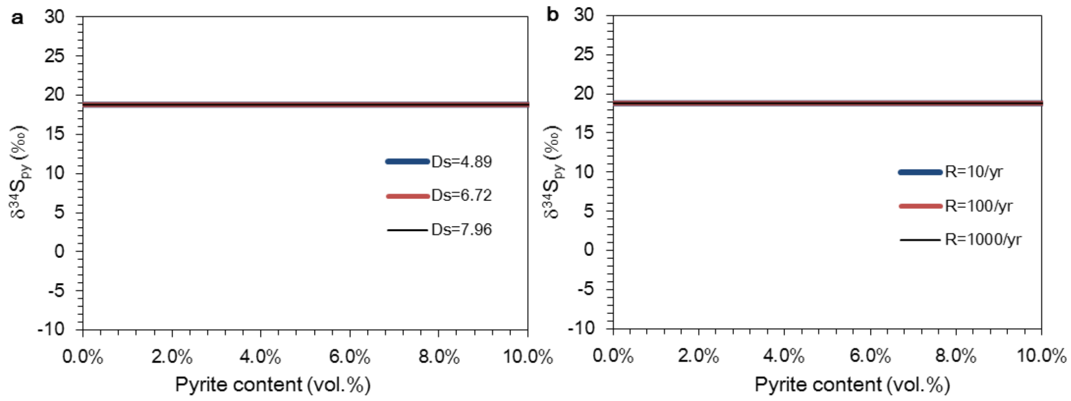

Supplementary Figure 10: Sensitivity test of the 1D-DR model. The default parameters for $\delta^{34}S_{H2S}$, Δ_{py} and $[H2S]_0$ are +20‰, 1‰ and 0.5 mM L^{-1}, respectively. (a): The $\delta^{34}S_{py}$ is not sensitivity to D_s when D_s varies between 4.89×10^{-6} cm^2 s^{-1} and 7.96×10^{-6} cm^2 s^{-1}. (b) The $\delta^{34}S_{py}$ is not sensitivity to R when R varies between 10 yr^{-1} and 1000 yr^{-1}.

Supplementary Table 1. Pyrite concretions content in the top of Nantuo Formation.

Section	Pyrite content(%)	Thickness(m)	Environment
Yazhai	5.8	10.1	Basin
Tongle	2.8	5	Basin
Silikou	7	9.5	Basin
Yangxi	6.2	8.1	Basin
Yuanjia	2	3.9	Basin
Huakoushan	2.1	4.5	Slope
Bahuang	2	2	Slope
Taoying	4.6	1.8	Slope
Siduping	3	0.5	Slope
Tianping	3.6	1.5	Slope
Songlin	0.1	0.1	Open shelf
Youxi	0.2	0.1	Open shelf
Huajipo	0.5	0.2	Open shelf

Supplementary Table 2. $\delta^{34}S$ of pyrite in the top of the Nantuo Formation in the Yangtze Block.

Sample NO.	$\delta^{34}S$	Pyrite Type	Section	Sedimentary environment
14SL-NT-2-2	10.1	concretion	Songlin	open shelf
14SL-NT-2	9.4	concretion	Songlin	open shelf
14SL-NT-1-2	8.1	concretion	Songlin	open shelf
14SL-NT-1-1	8.2	concretion	Songlin	open shelf
14SL-C-2	8.5	concretion	Songlin	open shelf
14SL-C-1-1	10.1	concretion	Songlin	open shelf
SL-1-1	12.2	concretion	Songlin	open shelf
SL-1-2	11.5	concretion	Songlin	open shelf
SL-1-3	11.5	concretion	Songlin	open shelf
SL-1-4	11.5	concretion	Songlin	open shelf
SL-1-5	11.2	concretion	Songlin	open shelf
SL-1-6	11.1	concretion	Songlin	open shelf
SL-1-7	11.1	concretion	Songlin	open shelf
SL-1-8	10.1	concretion	Songlin	open shelf
SL-2-1	10.4	concretion	Songlin	open shelf
SL-2-2	10.8	concretion	Songlin	open shelf
SL-2-3	11.0	concretion	Songlin	open shelf
SL-2-4	10.8	concretion	Songlin	open shelf
SL-2-5	11.1	concretion	Songlin	open shelf
SL-3-1	11.8	concretion	Songlin	open shelf
SL-3-2	11.8	concretion	Songlin	open shelf
SL-3-3	11.9	concretion	Songlin	open shelf
SL-3-4	11.7	concretion	Songlin	open shelf
SL-3-5	11.9	concretion	Songlin	open shelf
SL-4-1	12.3	concretion	Songlin	open shelf
SL-4-2	12.6	concretion	Songlin	open shelf
SL-4-3	11.3	concretion	Songlin	open shelf
SL-4-4	10.9	concretion	Songlin	open shelf
SL-4-5	10.8	concretion	Songlin	open shelf
SL-4-6	11.5	concretion	Songlin	open shelf
SL-4-7	11.1	concretion	Songlin	open shelf
SL-4-8	10.1	concretion	Songlin	open shelf
SL-4-9	12.2	concretion	Songlin	open shelf
SL-4-10	11.6	concretion	Songlin	open shelf
SL-4-11	12.1	concretion	Songlin	open shelf
SL-4-12	11.7	concretion	Songlin	open shelf
SL-4-13	11.8	concretion	Songlin	open shelf
SL-5-1	12.0	concretion	Songlin	open shelf

SL-5-2	11.7	concretion	Songlin	open shelf
SL-5-3	11.8	concretion	Songlin	open shelf
SL-5-4	11.9	concretion	Songlin	open shelf
SL-5-5	10.1	concretion	Songlin	open shelf
SL-5-6	12.4	concretion	Songlin	open shelf
YX-NT1-1	-2.5	concretion	Youxi	open shelf
YX-NT1-2	-3.1	concretion	Youxi	open shelf
YX-2-1	-5.0	concretion	Youxi	open shelf
YX-2-2	-4.3	concretion	Youxi	open shelf
YX-2-3	-2.7	concretion	Youxi	open shelf
YX-2-4	-4.2	concretion	Youxi	open shelf
YX-2-5	-2.7	concretion	Youxi	open shelf
YX-2-6	-4.0	concretion	Youxi	open shelf
YX-3-1	-3.0	concretion	Youxi	open shelf
YX-3-2	-4.5	concretion	Youxi	open shelf
YX-3-3	-3.7	concretion	Youxi	open shelf
YX-3-4	-5.5	concretion	Youxi	open shelf
YX-3-5	-3.4	concretion	Youxi	open shelf
YX-4-1	-5.4	concretion	Youxi	open shelf
YX-4-2	-4.4	concretion	Youxi	open shelf
YX-4-3	-3.8	concretion	Youxi	open shelf
YX-4-4	-5.2	concretion	Youxi	open shelf
YX-4-5	-4.3	concretion	Youxi	open shelf
14HKS-NT-4-2	26.6	concretion	Huakoushan	slope
14HKS-NT-4-1	16.1	concretion	Huakoushan	slope
14HKS-NT-3-2	30.9	concretion	Huakoushan	slope
14HKS-NT-3-1	33.7	concretion	Huakoushan	slope
14HKS-NT-2-2	23.8	concretion	Huakoushan	slope
14HKS-NT-2-1	23.4	concretion	Huakoushan	slope
14HKS-NT-1-2	27.5	concretion	Huakoushan	slope
14HKS-NT-1-1	27.2	concretion	Huakoushan	slope
HKS-6-1	18.5	concretion	Huakoushan	slope
HKS-6-2	18.6	concretion	Huakoushan	slope
HKS-6-3	19.1	concretion	Huakoushan	slope
HKS-6-4	24.4	concretion	Huakoushan	slope
HKS-6-5	36.1	concretion	Huakoushan	slope
HKS-6-6	27.5	concretion	Huakoushan	slope
HKS-6-7	37.1	concretion	Huakoushan	slope
HKS-6-8	16.4	concretion	Huakoushan	slope
HKS-6-9	16.9	concretion	Huakoushan	slope
HKS-6-10	16.9	concretion	Huakoushan	slope
HKS-7-1	29.6	concretion	Huakoushan	slope
HKS-7-2	27.2	concretion	Huakoushan	slope
HKS-7-3	27.0	concretion	Huakoushan	slope

HKS-7-4	36.1	concretion	Huakoushan	slope
HKS-7-5	29.7	concretion	Huakoushan	slope
HKS-7-6	31.0	concretion	Huakoushan	slope
HKS-8-1	28.8	concretion	Huakoushan	slope
HKS-8-2	29.6	concretion	Huakoushan	slope
HKS-8-3	31.8	concretion	Huakoushan	slope
HKS-8-4	31.0	concretion	Huakoushan	slope
HKS-8-5	27.6	concretion	Huakoushan	slope
HKS-8-6	29.9	concretion	Huakoushan	slope
HKS-9-1	26.6	concretion	Huakoushan	slope
HKS-9-2	26.5	concretion	Huakoushan	slope
HKS-9-3	26.2	concretion	Huakoushan	slope
HKS-9-4	27.5	concretion	Huakoushan	slope
HKS-9-5	26.3	concretion	Huakoushan	slope
HKS-9-6	26.2	concretion	Huakoushan	slope
BH3-1	26.5	concretion	Bahuang	slope
BH4-1	28.2	concretion	Bahuang	slope
BH4-2	21.0	concretion	Bahuang	slope
BH6-1	-5.0	disseminated	Bahuang	slope
BH6-2	-5.7	disseminated	Bahuang	slope
TP-45	13.7	disseminated	Taoying	slope
TP-46	20.5	disseminated	Taoying	slope
TP-47	40.1	disseminated	Taoying	slope
TP-48	34.7	disseminated	Taoying	slope
TP-49	29.2	disseminated	Taoying	slope
TP-50	21.9	disseminated	Taoying	slope
TP-51	39.1	disseminated	Taoying	slope
TP-52	28.4	disseminated	Taoying	slope
TP-53	19.4	disseminated	Taoying	slope
TP-54	25.0	disseminated	Taoying	slope
TP-55	26.4	disseminated	Taoying	slope
TP-57	48.1	disseminated	Taoying	slope
TL0-1	16.0	concretion	Tongle	basin
TL0-2	13.5	concretion	Tongle	basin
TL0-3	13.8	concretion	Tongle	basin
TL0-4	14.5	concretion	Tongle	basin
TL0-5	14.6	concretion	Tongle	basin
TL0-6	15.6	concretion	Tongle	basin
TL0-7	13.7	concretion	Tongle	basin
TL0-8	14.8	concretion	Tongle	basin
TL0-9	15.1	concretion	Tongle	basin
TL0-10	13.5	concretion	Tongle	basin
TL0-11	13.9	concretion	Tongle	basin
TL0-12	14.0	concretion	Tongle	basin

TL4-1	15.3	concretion	Tongle	basin
TL4-2	14.9	concretion	Tongle	basin
TL4-3	15.2	concretion	Tongle	basin
TL4-4	15.3	concretion	Tongle	basin
TL4-5	16.0	concretion	Tongle	basin
TL3-1	16.2	concretion	Tongle	basin
TL2-1	19.3	concretion	Tongle	basin
TL2-2	19.0	concretion	Tongle	basin
TL2-3	18.8	concretion	Tongle	basin
TL2-4	18.4	concretion	Tongle	basin
TL2-5	18.4	concretion	Tongle	basin
TL2-6	18.5	concretion	Tongle	basin
TL2-7	18.4	concretion	Tongle	basin
TL2-8	18.0	concretion	Tongle	basin
TL2-9	16.9	concretion	Tongle	basin
TL2-10	16.2	concretion	Tongle	basin
TL2-11	16.1	concretion	Tongle	basin
TL2-12	16.0	concretion	Tongle	basin
TL2-13	15.9	concretion	Tongle	basin
TL2-14	16.2	concretion	Tongle	basin
TL2-15	16.4	concretion	Tongle	basin
TL2-16	17.2	concretion	Tongle	basin
TL2-17	18.8	concretion	Tongle	basin
TL2-18	16.8	concretion	Tongle	basin
TL2-19	16.4	concretion	Tongle	basin
TL2-20	16.4	concretion	Tongle	basin
TL2-21	16.5	concretion	Tongle	basin
TL1-1	17.9	concretion	Tongle	basin
TL1-2	16.9	concretion	Tongle	basin
TL1-3	17.9	concretion	Tongle	basin
TL1-4	17.9	concretion	Tongle	basin
TL1-5	17.7	concretion	Tongle	basin
TL1-6	18.1	concretion	Tongle	basin
TL1-7	18.2	concretion	Tongle	basin
TL1-8	18.1	concretion	Tongle	basin
TL1-9	16.8	concretion	Tongle	basin
TL1-10	17.0	concretion	Tongle	basin
TL1-11	18.0	concretion	Tongle	basin
TL1-12	17.7	concretion	Tongle	basin
TL1-13	17.2	concretion	Tongle	basin
TL1-14	16.8	concretion	Tongle	basin
TL1-15	13.8	concretion	Tongle	basin
TL1-16	15.2	concretion	Tongle	basin
TL1-17	18.1	concretion	Tongle	basin

YZ1-1	22.8	concretion	Yazhai	basin
YZ1-2	22.3	concretion	Yazhai	basin
YZ1-3	21.0	concretion	Yazhai	basin
YZ1-4	20.5	concretion	Yazhai	basin
YZ1-5	18.7	concretion	Yazhai	basin
YZ1-6	18.9	concretion	Yazhai	basin
YZ1-7	16.7	concretion	Yazhai	basin
YZ1-8	19.7	concretion	Yazhai	basin
YZ1-9	19.7	concretion	Yazhai	basin
YZ1-10	17.8	concretion	Yazhai	basin
YZ1-11	19.9	concretion	Yazhai	basin
YZ1-12	21.5	concretion	Yazhai	basin
YZA1-1	22.2	concretion	Yazhai	basin
YZA1-2	19.0	concretion	Yazhai	basin
YZA1-3	21.4	concretion	Yazhai	basin
YZA1-4	13.6	concretion	Yazhai	basin
YZA1-5	21.8	concretion	Yazhai	basin
YZA1-6	21.8	concretion	Yazhai	basin
YZA1-7	19.1	concretion	Yazhai	basin
YZA1-8	21.1	concretion	Yazhai	basin
YZA1-9	21.0	concretion	Yazhai	basin
YZA1-10	19.4	concretion	Yazhai	basin
YZA1-11	20.0	concretion	Yazhai	basin
YZA1-12	21.5	concretion	Yazhai	basin
YZ2-1	20.1	concretion	Yazhai	basin
YZ2-2	19.4	concretion	Yazhai	basin
YZ2-3	21.6	concretion	Yazhai	basin
YZ2-4	19.4	concretion	Yazhai	basin
YZ2-5	18.9	concretion	Yazhai	basin
YZ3-1	21.5	concretion	Yazhai	basin
YZ3-2	19.9	concretion	Yazhai	basin
YZ3-3	21.5	concretion	Yazhai	basin
YZ3-4	21.5	concretion	Yazhai	basin
YZ4-1	14.5	concretion	Yazhai	basin
YZ4-2	14.8	concretion	Yazhai	basin
YZ4-3	16.0	concretion	Yazhai	basin
YZ4-4	15.9	concretion	Yazhai	basin
YZ4-5	15.5	concretion	Yazhai	basin
YZ4-6	17.7	concretion	Yazhai	basin
YZ5-1	16.5	concretion	Yazhai	basin
YZ5-2	14.4	concretion	Yazhai	basin
YZ5-3	19.6	concretion	Yazhai	basin
YZ5-4	21.6	concretion	Yazhai	basin
YZ5-5	23.7	concretion	Yazhai	basin

YZ5-6	19.3	concretion	Yazhai	basin
TP-108-3	16.5	concretion	Yuanjia	basin
TP-108-4	19.6	concretion	Yuanjia	basin
TP-108-5	19.8	concretion	Yuanjia	basin
TP-108-6	19.7	concretion	Yuanjia	basin
TP-108-1	19.5	concretion	Yuanjia	basin
TP-109	19.5	concretion	Yuanjia	basin
TP-110	19.3	concretion	Yuanjia	basin
TP-111-1-1	19.7	concretion	Yuanjia	basin
TP-111-1-1	16.8	concretion	Yuanjia	basin
TP-111-1-2	17.9	concretion	Yuanjia	basin
TP-111-1-3	19.6	concretion	Yuanjia	basin
TP-111-1-4	18.6	concretion	Yuanjia	basin
TP-111-1-5	17.7	concretion	Yuanjia	basin
TP-111-1-6	17.5	concretion	Yuanjia	basin
TP-111-1-7	18.3	concretion	Yuanjia	basin
TP-111-2-1	17.9	concretion	Yuanjia	basin
TP-111-2-2	18.6	concretion	Yuanjia	basin
TP-111-2-3	18.4	concretion	Yuanjia	basin
TP-111-2-4	19.2	concretion	Yuanjia	basin
TP-112-1-1	19.4	concretion	Yuanjia	basin
TP-112-1-2	19.2	concretion	Yuanjia	basin
TP-112-1-3	19.3	concretion	Yuanjia	basin
TP-112-1-4	17.6	concretion	Yuanjia	basin
TP-112-1-5	18.7	concretion	Yuanjia	basin
TP-112-1-6	16.1	concretion	Yuanjia	basin
TP-112-1-7	15.7	concretion	Yuanjia	basin
TP-112-1-8	18.5	concretion	Yuanjia	basin
TP-112-2-1	19.1	concretion	Yuanjia	basin
TP-112-2-2	18.1	concretion	Yuanjia	basin
TP-112-2-3	19.9	concretion	Yuanjia	basin
TP-112-2-4	17.5	concretion	Yuanjia	basin

Supplementary Table 3. Multiple sulfur isotopes of pyrite in the top of the Nantuo Formation in the Yangtze Block.

Sample No.	$\delta^{33}S$	$\delta^{34}S$	$\delta^{36}S$	$\Delta^{33}S$	$\Delta^{36}S$	Section	Sedimentary environment
YX-1	-0.6	-1.4	-3.0	0.080	-0.340	Youxi	open shelf
YX-2	-0.5	-1.2	-2.6	0.098	-0.238	Youxi	open shelf
SL-1	6.0	11.5	22.0	0.030	-0.001	Songlin	open shelf
SL-2	5.9	11.4	21.7	0.018	-0.074	Songlin	open shelf
SL-3	6.3	12.2	23.2	0.059	-0.078	Songlin	open shelf
TP-49	14.7	28.7	55.4	-0.005	0.263	Taoying	slope
TP-53	10.8	21.0	40.5	-0.010	0.203	Taoying	slope
TP-54	11.6	22.6	43.1	0.056	-0.210	Taoying	slope
HK5-2-1	14.5	28.4	54.4	0.038	-0.139	Huakoushan	slope
HK5-2-2	7.8	15.0	28.3	0.067	-0.416	Huakoushan	slope
HK5-8-1	13.2	25.8	49.2	0.053	-0.317	Huakoushan	slope
HK5-8-2	15.5	30.2	58.1	0.021	-0.126	Huakoushan	slope
HK5-8-3	13.8	26.9	51.4	0.036	-0.289	Huakoushan	slope
YZ-1-1	8.8	17.2	32.9	-0.002	-0.048	Yazhai	basin
YZ-1-2	10.6	20.7	39.7	0.013	0.002	Yazhai	basin
YZ-3	12.3	24.1	46.7	0.018	0.435	Yazhai	basin
YZ-4-1	8.4	16.3	30.9	0.020	-0.238	Yazhai	basin
YZ-4-2	8.4	16.2	30.8	0.028	-0.271	Yazhai	basin
TL-1-1	9.7	18.9	35.7	0.072	-0.421	Tongle	basin
TL-1-2	9.1	17.6	33.2	0.070	-0.468	Tongle	basin
TL-2-1	9.0	17.4	32.9	0.068	-0.343	Tongle	basin
TL-2-2	9.3	18.1	34.2	0.068	-0.382	Tongle	basin
TL-2-3	9.9	19.3	36.6	0.043	-0.331	Tongle	basin
TL-4-1	8.5	16.5	31.3	0.051	-0.374	Tongle	basin
TL-4-2	7.7	14.9	28.2	0.049	-0.362	Tongle	basin
TP-108-1	9.9	19.4	37.0	-0.005	-0.081	Yuanjia	basin
TP-108-3	9.4	18.4	35.1	0.013	-0.117	Yuanjia	basin
TP-108-5	10.2	20.0	38.6	-0.008	0.293	Yuanjia	basin
TP-112-1	10.6	20.7	39.6	-0.001	-0.078	Yuanjia	basin
TP-112-3	10.5	20.5	39.2	-0.003	-0.062	Yuanjia	basin
TP-112-7	10.4	20.4	39.0	-0.001	-0.084	Yuanjia	basin

Supplementary Table 4. List of parameters used in the 1D-DAR model.

**Parameter**	**Default value**	**Reference**	**Value for sensitivity test**
D_s	3.61×10^{-6} cm^2 s^{-1}	8	2.29×10^{-6}–4.92×10^{-6} cm^2 s^{-1}
s	0.01 cm yr^{-1}	8	0.005–0.2 cm yr^{-1}
R	1 yr^{-1}	10	0.1 yr^{-1}–10 yr^{-1}
$[SO4]_0$	3 mM L^{-1}	7	0.5–5mM L^{-1}
$\delta^{34}S_{sw}$	+30‰	9	
α	0.96	10	

Supplementary Table 5. List of parameters used in the Rayleigh distillation model.

Parameter	Value	Reference
$\delta^{34}S_{sw}$	+30‰	9
α	0.96–0.98	10
Φ	60%	8
[SO4]	3 mM/L	7
f	0 to 1	

Supplementary Table 6. List of parameters used in the 1D-DR model.

**Parameter**	**Value**
D_s	4.89×10^{-6}–$7.96 \times 10^{-6} cm^2\ s^{-1}$
R	10–1000 yr^{-1}
$[HS]_0$	0.5 mM L^{-1}
$\delta^{34}S_{H2S}$	-5‰ to +30‰
Δ_{py}	1 ‰

Supplementary references

1 Condon, D. *et al.* U-Pb ages from the Neoproterozoic Doushantuo Formation, China. *Science* **308**, 95–98 (2005).

2 Zhang, S., Jiang, G. & Han, Y. The age of the Nantuo Formation and Nantuo glaciation in South China. *Terra Nova* **20**, 289–294 (2008).

3 Zhang, Q. R., Chu, X. L. & Feng, L. J. Chapter 32 Neoproterozoic glacial records in the Yangtze Region, China. *Geological Society, London, Memoirs* **36**, 357–366 (2011).

4 Jiang, G., Shi, X., Zhang, S., Wang, Y. & Xiao, S. Stratigraphy and paleogeography of the Ediacaran Doushantuo Formation (ca. 635–551Ma) in South China. *Gondwana Research* **19**, 831–849 (2011).

5 Wang, J. & Li, Z. X. History of Neoproterozoic rift basins in South China: implications for Rodinia break-up. *Precambrian Research* **122**, 141–158 (2003).

6 Hurtgen, M. T., Arthur, M. A. & Halverson, G. P. Neoproterozoic sulfur isotopes, the evolution of microbial sulfur species, and the burial efficiency of sulfide as sedimentary pyrite. *Geology* **33**, 41-44 (2005).

7 Huang, J. *et al.* The sulfur isotope signatures of Marinoan deglaciation captured in Neoproterozoic shallow-to-deep cap carbonate from South China. *Precambrian Research* **238**, 42–51, doi:10.1016/j.precamres.2013.09.002 (2013).

8 D., S. H. & M., Z. Marine Geochemistry. *Springer, Germany*, 80–81 (2006).

9 Crockford, P. W. *et al.* Triple oxygen and multiple sulfur isotope constraints on the evolution of the post-Marinoan sulfur cycle. *Earth and Planetary Science Letters* **435**, 74–83, doi:10.1016/j.epsl.2015.12.017 (2016).

10 Canfield, D. E. Isotope fractionation by natural populations of sulfate-reducing bacteria. *Geochimica et Cosmochimica Acta* **65**, 1117–1124, doi:https://doi.org/10.1016/S0016-7037(00)00584-6 (2001).
